# ‘Looking before and after’: Can simple eye tracking patterns distinguish poetic from prosaic texts?

**DOI:** 10.3389/fpsyg.2023.1066303

**Published:** 2023-01-26

**Authors:** Rhiannon Corcoran, Christophe de Bezenac, Philip Davis

**Affiliations:** ^1^Department of Primary Care and Mental Health, Institute of Population Health, University of Liverpool, Liverpool, United Kingdom; ^2^Department of Molecular and Clinical Pharmacology, Institute of Translational Medicine, University of Liverpool, Liverpool, United Kingdom

**Keywords:** Literary reading, regressive eye movements, fixations, semantic reappraisal, poetry

## Abstract

**Introduction:**

The study of ‘serious’ literature has recently developed into an emerging field called neurocognitive poetics that applies cognitive neuroscientific techniques to examine how we understand and appreciate poetry. The current research used eye-tracking techniques on a small sample of young adults to see if and how the reading of short pieces of poetry differed from the reading of matched prosaic texts.

**Methods:**

With ‘proof of concept’ intentions reflecting arguments first proposed by 19th Century literary figures, there was a particular focus on the differences between the reading of poetry and prose in terms number and frequency of fixations and regressive eye movements back and forth within the texts in this two-by-two experimental design (poetry vs. prose x need vs. no need for final line reappraisal).

**Results:**

It was found that poetic pieces compared to prosaic pieces were associated with more and longer fixations and more regressive eye movements throughout the text. The need to reappraise meaning at the prompt of a final line was only significantly associated with more regressive eye movements. Comparisons examining the 4 text conditions (poetic reappraisal, poetic non-reappraisal, prosaic reappraisal, and prosaic non-reappraisal) showed that the poetic reappraisal condition was characterised by significantly more regressive eye movements as well as longer fixations compared to the prosaic non-reappraisal condition. No significant correlations were found between self-reported literary familiarity and eye tracking patterns.

**Discussion:**

Despite limitations, this proof-of-concept study provides insights into reading patterns that can help to define objectively the nature of poetic material as requiring slower reading particularly characterised by more and longer fixations and eye movements backwards through the texts compared to the faster, more linear reading of prose. Future research using these, and other psychophysiological metrics can begin to unpack the putative cognitive benefits of reading literary material.

## Introduction

Wolf (2018) writes of the reading brain’s connectedness, in acts of complex literary reading: ‘At least as many things are happening in zigzagging, feed-forward, feed-backward interactivity as are occurring linearly.’

Wolf’s idea, that literary reading, in comparison with more literal forms of processing, engages deeper and wider potential of the human mind, has a history. In contrast to what we might call reading as a simple information gathering exercise involving the sequential left-to-right scanning of text, the Romantic poet and philosopher Samuel Coleridge (1817) spoke of literary reading as developing movements and relations of mind that were not simply linear or literally straight-forward:

“*Most of my readers will have observed a small water-insect on the surface of rivulets … and will have noticed, how the little animal wins its way up against the stream, by alternate pulses of active and passive motion, now resisting the current, and now yielding to it in order to gather strength and a momentary fulcrum for a further propulsion. This is no unapt emblem of the mind’s self-experience in the act of thinking. There are evidently two powers at work, which relatively to each other are active and passive; and this is not possible without an intermediate faculty, which is at once both active and passive. In philosophical language, we must denominate this intermediate faculty in all its degrees and determinations, the IMAGINATION*. (Biographia Literaria, Chapter 7)

In the act of literary reading, as a model for what Coleridge takes to be the deepest human thinking, the creation of this intermediate state or middle zone between active and passive depends on the reader not being “*carried forward by merely mechanical impulse*.” Instead, processing is more a ‘to-and fro-motion’: “*At every step the reader pauses and half recedes, and from the retrogressive movement collects the force which again carries him onward*” (Coleridge, 1817).

Coleridge’s collaborator, Wordsworth (1805) asserted this understanding in his preface to their jointly published *Lyrical Ballads*, emphasising the importance of a closer future relation between art and science, concluding:

“*Emphatically may it be said of the Poet, as Shakespeare hath said of man,” “that he looks before and after.*”

It is argued that literary reading involves the immersion of attention in a dense medium of thick description which holds back over-speedy decisions based on habitual biases, enabling it to process this more complex material for deeper understanding. This processing style likely involves ‘*the mind’s self-experience of the act of thinking*’ (Wordsworth, 1805) carefully following the journey of lines and sentences unfolding enroute, without advance knowledge of the final meaning. So, it is claimed that while literary reading is ‘slow reading’ compared to scanning, by being slow, it exercises the mobility and flexibility of an actively thinking mind in contrast to a mind more simply engaged in the act of information gathering. It is thought that this deeper form of reading channels self-reflection, critical analysis, inductive and deductive reasoning all together during the reading process, creating the mix of a contemplative mind (Wolf and Barzillai, 2009).

The largely implicit processes of sentence comprehension that require internal representation of the syntax and the construction of meaning from it have been reviewed by Staub (2015). In particular, Staub focuses on how physiological monitoring through eye tracking and Event Related Potentials can help to uncover the components of this invisible processing where the issue of serial versus parallel processing continues to be an active area of research. In contrast to serial processing, for parallel processing models, the number of ambiguities or uncertainties reflected in a piece of text increases its computational difficulty. The ambiguity that characterises poetry which requires slow or deep reading (Wolf, 2018) implies a parallel processing model for this type of reading, although so-called hybrid models can also account for the inferred differences in reading of literary compared to more prosaic texts (e.g., Van Gompel et al., 2005). Furthermore, the ambiguity characteristic of haiku texts has been related to particular individual and cultural differences such as emotionality and mental imagery skills (Hitsuwari and Nomura, 2022a,b) The demands placed on verbal working memory is also related to the complexity of the sentence and there are debates about whether a specialised system has developed to tackle these unique processing demands (Staub, 2015). However, there seems to be little specific focus on how the reader’s autobiographical recollections impact the achievement and nature of understanding as we read. The engagement of these processes are inferred in Wolf and Barzillai’s (2009) idea of deep reading where inductive or analogical processes are invoked. Certainly, the uniqueness of life experiences will influence how we each interpret other’s stories in real life and as reflected in literary material.

While fMRI has been quite extensively used to explore meaning derivation from text, eye tracking has been used less frequently for this purpose. Assuming that what is attended to will be what is processed (Majaranta and Bulling, 2014), eye tracking data can provide a clear, moment-to-moment indication of a reader’s information seeking strategies. In turn, this information permits tentative inferences about readers’ emotional and cognitive engagement with the written word. Eye-tracking studies of reading have tended typically to examine low level visual and lexical processing of text. However, eye movements are also influenced by higher order comprehension and the processes of meaning derivation (e.g., Birch and Rayner, 1997; Cook and Myers, 2004; Rayner et al., 2006).

Linguistic and cognitive processing during reading arises at single word, sentence level and at whole text level. According to Jarodzka and Brand-Gruwel’s (2017) model of real-world reading, at the single word and sentence level key questions answered using eye tracking are about when and where we fixate or skip words. At the whole text level, eye tracking can usefully indicate which parts of a text we re-visit or process more and which part(s) we scan. As such, these different levels refer, respectively, to ocular motor control and linguistic processing during reading versus reading comprehension. At both levels, fixations on words and the number of regressive eye movements are variables of interest with both being indicative of perceived text difficulty and therefore regarded to be under cognitive control. It is generally agreed that both fixations and regressions are sensitive to word frequency, difficulty and unpredictability (Jarodzka and Brand-Gruwel, 2017).

Importantly, while most reading happens in forward motion, backward movements from one word to another are not that uncommon with Rayner and Pollatsek (1989) showing that between 10 and 25% of eye movements are backwards within a text. These regressive eye movements appear integral to comprehension as they reflect a revisiting of words to reprocess information towards the final stage of comprehension, the building of a situational model. A useful situational model brings a congruence of local context (the text) and existing real-world comprehension (e.g., McNamara et al., 1996).

With regard to comprehension of whole texts, research has shown there to be differences between styles of reading and the number of fixations and regressions (Reichle et al., 2010; Schotter et al., 2014). Thus, the purpose of the reading task and the nature of the reading material itself influences how people read as reflected in duration and frequency of fixations and regressions (Ehrlich and Rayner, 1981; Rayner and Duffy, 1986). Furthermore, the nature of the reading material and the purpose for reading it interact with individual reader differences (Jarodzka and Brand-Gruwel, 2017) ranging from specific learning difficulty, prior knowledge and experience of the world to topic or material expertise (e.g., Reingold and Sheridan, 2011). These individual differences further influence how our cognitive resources are distributed across texts through top-down influences on eye movements.

Neuroscience devoted to the study of the processing of literary texts has been referred to as neurocognitive poetics (Jacobs, 2015). In an early example of this emerging field, a study using the same stimuli as used here, O’Sullivan et al. (2015) examined the processing and derivation of meaning of short segments of complex text which were either poetic or prosaic and which either did or did not require substantial reappraisal resulting from a final surprising line. One of O’Sullivan et al.’s findings was an association between the recognition of poetic texts and activation of the right dorsal caudate, an area associated with tolerance of ambiguity or uncertainty. Thus, the authors argued that engagement with literary texts such as poetry has potential to alter thinking styles in a way that will benefit mental health and wellbeing, encouraging fluidity in the consideration of alternative meanings and valuing, instead of fearing, uncertainty. As we live day-to-day in an uncertain world where, for example, others’ minds must be modelled and responded to quickly and accurately, this is a valuable form of learning experience (Corcoran and Oatley, 2019). The complex texts of literature, dealing as they so often do with existential, human issues, are defined by the need of the reader to take a layered perspective of possibilities within an unfolding narrative while bearing in mind protagonists’ stances beliefs and intentions. Therefore, it is suggested that a growing literary awareness, emerging from the experience of literary reading, has potential, to support fuller, more engaged life experiences.

In a study interested in the issue of the need to reappraise information, Müller et al. (2017) used eye tracking during the reading of English language haikus (ELH). Their findings supported the suggestion that processes of meaning construction are reflected in patterns of eye movements during reading as well as re-reading. Furthermore, the eye movement patterns seen in ELHs requiring re-appraisal were more complex, suggestive of greater effort to reach meaning, compared to patterns seen for the ELH that did not involve re-appraisal.

Considering the relative infancy of neurocognitive poetics, the current study aimed to explore the slow literary reading mind using simple eye tracking metrics. We were interested to uncover the mental to-and fro movement thought to define the literary reading style and which psychologists and neuroscientists would describe as cognitive reappraisal or meaning derivation processes. It attempted to go beyond Müller et al.’s (2017) analyses by comparing poetic and prosaic pieces and further, by investigating the reading pattern that identifies the need for re-appraisal across stimuli pieces. Thus, the present study explored eye tracking patterns, indicative of information processing, of poetic versus prosaic segments of texts half of which embedded the need to revise one’s understanding at the final line. The inclusion of this variable enabled us to recognise more readily the effects of major reappraisal reflecting semantic model updating on eye movement patterns.

We aimed to explore the frequency and duration of fixations and the number of long-range regressions (backward eye movements demonstrating a revisiting of earlier text) as measures of the need for more detailed and effortful information processing (Mitchell et al., 2008; Rayner, 2009). We predicted that poetic compared to prosaic texts and texts that explicitly required re-appraisal of meaning at the last line would be associated with eye tracking patterns indicative of the slower literary reading that Coleridge and Wordsworth described in the early 1800s. We anticipated the extent of this reading style to be associated with familiarity with literary material. Specifically, the predictions were:

1. Poetic pieces would require more regressive eye movements and longer and more fixations than prosaic pieces.
2. Pieces that required a major reappraisal of meaning at the point of the final line would be characterised by more regressive eye movements and longer and more fixations than pieces that did not require major reappraisal at the end.
3. Participants’ reported familiarity with literary material would be associated with fixations frequency and duration and number of regressive eye movements. However, the direction of the expected correlation was unclear as none of the participants were literary experts. On the one hand, level of familiarity might be associated with easier reading of the material, reflecting greater cognitive assurance with the texts. On the other hand, greater familiarity may prompt a greater recognition of the need to dwell on and revisit words deemed to be of literary value.

## Method

### Participants

Twenty-seven UG student participants were recruited into this study with 16 (13 females) producing complete eye-tracking data for all stimuli of sufficient quality to enable full analysis. Reasons for exclusion of data from 11 participants included, not consistently reading the stimuli on time, failure of the eye-tracking procedure and procedural human error. The 16 participants whose complete data sets were used had a mean age of 19.9 (+/−1.1) and were all native English speakers who did not study, nor claim specific expertise, in literature. All had normal uncorrected vision and none declared a specific learning difficulty. Informed written consent was collected from all participants in accordance with the University Research Ethics Committee processes.

### Stimuli

Experimental stimuli were a subset of the original 48 stimuli behaviourally validated by O’Sullivan et al. (2015) using a sample of 30 individuals ranging in age between 16 and 65 and coming from a mix of educational backgrounds. Using 7-point scales, each stimulus was rated by the sample in terms of confidence that meaning had been understood; the feeling generated by them (negative-mixed-positive affect); the extent to which each had an expected or unexpected final meaning; and how poetic each was felt to be. Balancing the need to avoid undue fatigue with collecting adequate data for analysis, 18 poetic and 18 prosaic stimuli (9 with expected and 9 with unexpected final meanings) were selected for use in this study based on the behavioural ratings collected by O’Sullivan et al. ensuring that the number of words per piece did not significantly differ between poetic and prosaic conditions and that valence was matched across conditions. The stimuli were published, but not widely familiar 4-line poetic pieces (P) chosen by the literature scholars on the research team. Prosaic control pieces (C) were constructed by team members to match the poetic texts on parameters including: word count, punctuation, linguistic complexity, valence and global theme content. Poetic and prosaic pieces were further subdivided into those that gave rise to a final global meaning that was unexpected based on the first 3 lines of the text, promoting sematic reappraisal (referred to as the reappraisal condition), and those with a consistent linearly emerging global meaning requiring no reappraisal (referred to as the non-reappraisal condition). Two examples of each stimuli type are provided in Table 1. Further information about the stimuli including validation procedures can be found in O’Sullivan et al. (2015). In summary, the 2 × 2 design comprised conditions: poetic reappraisal; poetic non-reappraisal; prosaic reappraisal; prosaic non-reappraisal.

**Table 1:** Examples of text stimuli by condition (N.B. the whole stimuli set is available from the authors by request).

Stimuli condition	Text example
Poetic reappraisal	“Do you think of me as I think of you, My friends, my friends?” – She said it from the sea.
	It seemed not much to ask and yet too much
	Is this “Think of me as I think of you”?
Poetic reappraisal	She lived unknown, and few could know
	When Lucy ceased to be,
	But she is in her grave, and, oh,
	The difference to me
Prosaic reappraisal	“I do not know what you are thinking” she said
	She was also unsure what he thought of her.
	Hoping that this would prompt him
	She said “I think about you a lot.”
Prosaic reappraisal	She lived a lonely life in the country
	Where he tried to find her,
	When he saw the bright and lively house,
	He knew she was dead
Poetic non-reappraisal	Rain, midnight rain, nothing but the wild rain
	On this bleak hut, and solitude, and me,
	Remembering again that I shall die
	And neither hear the rain nor give it thanks
Poetic non-reappraisal	You cannot move for memories in here
	Tripped up, nudged, shins barked in the dark
	Against the sharp and unexpected corners
	Of the other days
Prosaic non-reappraisal	I had an awful dream the other night.
	It was pouring with rain and I was all alone
	On a remote and deserted island
	And I knew I was going to die out there.
Prosaic non-reappraisal	I have searched everywhere
	Looked in all the dark and dusty corners
	In my house and in my mind too
	But I just cannot find my old photo album

### Eye-tracking and stimuli presentation

We recorded eye movement data using a monocular (right eye) head-mounted Pupil Labs eye tracker connected to a computer.^1^ The devise, worn like a pair of glasses, includes an eye camera directed at the participant’s eye (IR global shutter camera, 400×400, 120 hz, latency = 4.5 ms) and a world camera focusing on the stimuli displayed in front of the participant (100-degree wide angle lens, 1,280×720, 60 hz, latency = 5.7 ms). With Pupil Labs’ software Capture, the shape and orientation of pupils are computed with contour-detection algorithms (Kassner et al., 2014) and mapped to visual scene coordinates based on calibration parameters.

Text stimuli were presented to participants on a HP Compaq LA2306x computer screen (LED-backlit, LCD, aspect ratio = 16:9, 1920 × 1,080, 60 hz) connected to a MacBook Air (10.12.6), which was used to run the eye tracking software and stimulus presentation program written in Pure Data^2^ – a visual programming language for real-time multimedia processing.

### Procedure

All participants completed the task in a quiet 2 × 3 m cubicle with controlled artificial lighting consistent across eye-tracking sessions. Participants were seated approximately 0.5-1 m away from the computer screen, adjusted so that the centre of the screen was at eye level. Participants were given written and verbal instructions and then fitted with the headset. Camera adjustments were made to best capture each participant’s right eye and then the eye tracking system was screen calibrated.

Following successful calibration, participants were asked to read the 36 texts in a randomised order. To standardise the starting location of their gaze, all participants were asked to look at a fixation cross prior to each text. Participants were told to read each text until they understood it and then, using the index finger of their dominant hand, to press the spacebar on the Macbook keyboard to move on to the next stimulus. Participants were made aware that each text would be shown for a maximum of 25 s and that they should try to respond within that timeframe if possible. All included participants met this requirement for all texts.

After completing the eye-tracking task, participants were asked three questions to assess their familiarity with literary material. These questions were (1) How often do you read poetry (0 = never; 5 = very often); (2) how often do you write poetry and/or song lyrics (0 = never; 5 = very often); and (3) how good do you think you are at reading and understanding poetry and complex literature (0 = not all, 5 = very good). This produced a subjective literary familiarity score out of 15.

### Eye movement analysis

Fixations are characterised by a series of gaze points that occur in close time and range, resulting in a gaze cluster. We identified fixations using a dispersion-based algorithm (I-DT). As fixations are typically at least between 100 and 200 ms in duration, we included a minimum duration threshold of 150 ms in accordance with previous work (Salvucci and Goldberg, 2000). To identify regressions, areas of interest (AOIs) were first created for each word in each text using optical character recognition on stimuli images. Words within texts were then numbered sequentially (irrespective of line position). Most regressions during reading are made to the immediately preceding word, but this short-range backward motion tends to be related to low level comprehension factors (Rayner, 2009). Therefore, it was decided to focus on backward regressive eye movements of greater than one word back for two main reasons. First, research suggests that so-called immediate regressions (back to the immediately preceding word) can be influenced, in large part, by ocular motor accuracy. Second, immediate regressions also reflect the tendency in word level scanning to automatically skip some words (Jarodzka and Brand-Gruwel, 2017). As neither ocular motor accuracy nor automatic scan reading style were of interest in this research, it was decided to ignore the forms of regressive eye movements most associated with them. Instead, consistent with the intention to extract indices widely regarded to reflect focused reading (Reichle et al., 2010), text comprehension and meaning-making, we analysed only regressive eye movements of at least two words back.

## Results

**Figure 1:**
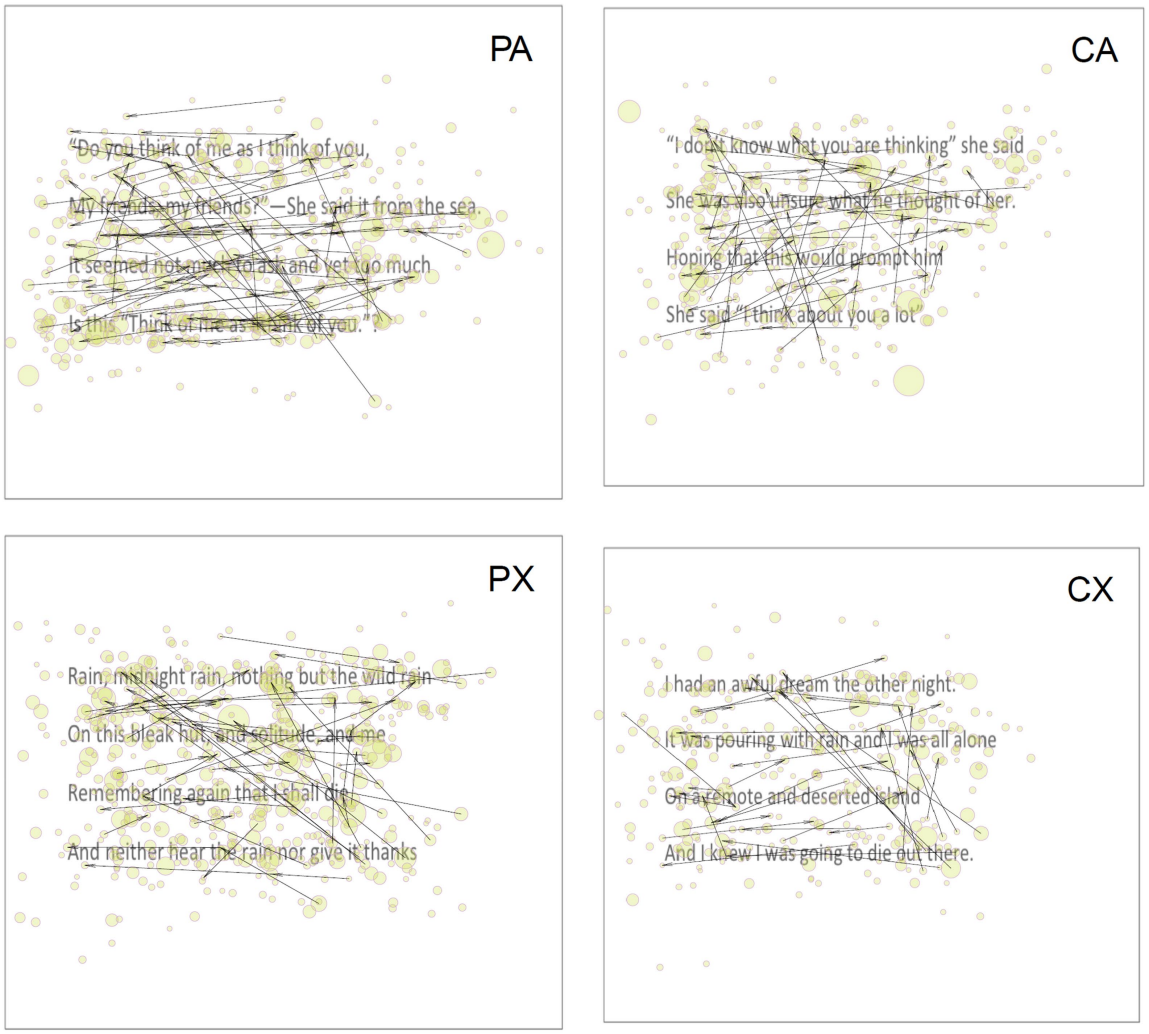
Example fixations and revisits/regressions by stimuli type. PA, poetic non-re-appraisal; CA, prosaic non-reappraisal; PX, poetic reappraisal; CX, prosaic reappraisal.

The data collected was a mix of normally and non-normally distributed data. As attempts to normalise the non-normally distributed data using log and square root transformation were unsuccessful, non-parametric analyses were carried out where appropriate. Descriptive statistics, divided according to text type (poetic, prosaic, reappraisal, non-reappraisal), are reported in Table 2 where the statistics reported reflect the distribution of the data. Figure 1 provides example texts showing regressive eye movements and fixation points with point size indicative of fixation duration.

**Table 2:** Descriptive statistics for the eye tracking variables divided according to stimuli type.

Eye tracking measure	Poetic stimuli	Prosaic stimuli	Reappraisal prompted by 4th line	No reappraisal prompted by 4th line
Number of revisits/regressions	65 (143)	47 (84)	65.61, 9.09	56.06, 6.72
Number of fixations	101 (88)	66.5 (87)	89.44 +/−28.13 7.03	86.75 +/−25.76 6.44
Duration of fixations (secs)	0.54 +/−0.26 0.065	0.52 +/−0.27 0.067	0.54 +/−0.29 0.072	0.53 +/−0.25 0.062

Median (Range); Mean, Standard Error as appropriate.

Outlier analyses were conducted on data for total regressive eye movements, total number of fixations and total duration of fixations across the stimuli types. Using the 1.5× interquartile range (IQR) and the 3× IQR rules, one potential outlier in the data relating to each of total regressions and total duration of fixations was identified when the 1.5 IQR rule was used. No outliers were identified when the 3× IQR rule was applied, however. As Hoaglin and Inglewicz (1987) argued that the 1.5 IQR rule may be too stringent, wrongly identifying outliers, the results of the 3× IQR rule were accepted for this proof of concept study.

### Text type – Poetry versus prose

As the regressive eye movement and fixation data was not normally distributed, a series of Wilcoxon signed ranks tests showed that there was a significant difference in the number of regressions between poetic and prosaic pieces (*T* = 16, *p* < 0.01, *r* = −0.63, one-tailed); a significant difference in the number of fixations between poetic and prosaic pieces (*T* = 2, *p* < 0.001, *r* = −0.82 one tailed) and a significant difference in the duration of fixations between poetic pieces and prosaic pieces (*T* = 14, *p* < 0.005, *r* = −0.70, one-tailed). For all these eye-tracking metrics, poetic pieces outnumbered prosaic pieces.

### Reappraisal versus non-reappraisal of meaning

To examine the impact of the need for major reappraisal forced by the final line of the texts paired samples t-tests were used. Analysis showed that the reading of reappraisal pieces was characterised by significantly more regressive eye movements than the reading of non-reappraisal pieces [*t* (15) = 2.40, *p* < 0.05, *r* = 0.53, one-tailed]. However, a paired samples t-tests showed no significant difference in the number of fixations between reappraisal pieces and non-reappraisal pieces, [*t* (15) = 0.72, *p* = 0.242, one-tailed] while a Wilcoxon sign ranked test showed no difference in the duration of fixations between reappraisal and non-reappraisal pieces [*T* = 42, *p* = 0.096, one-tailed]. These analyses demonstrate that the nature of the final line affected only the need to revisit preceding text.

### Text type by need to reappraise

**Figure 2:**
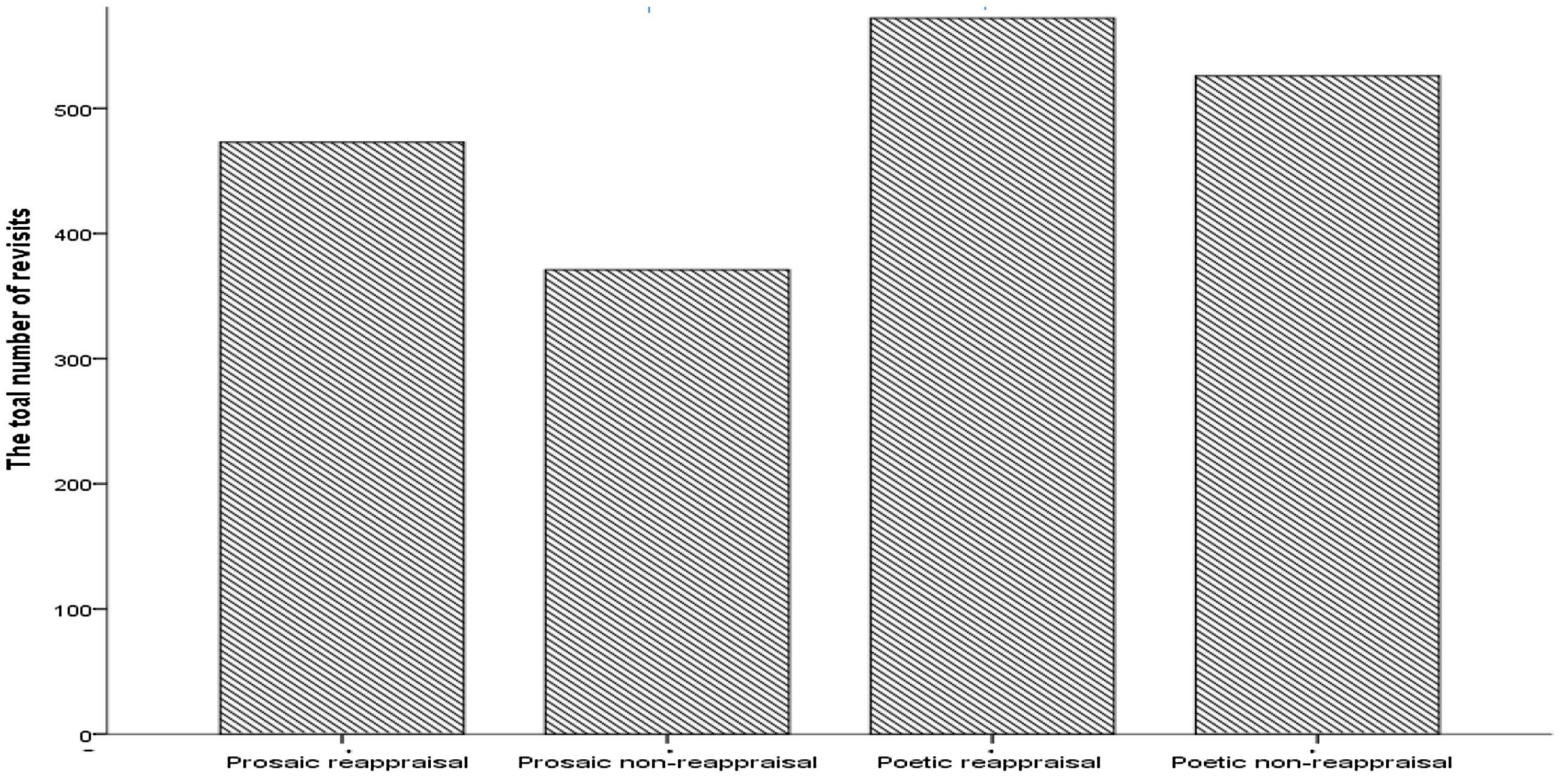
Bar graph showing the total number of regressive eye movements by text type. Poetic reappraisal > prosaic non-reappraisal; *p* = 0.002.

A significant non-parametric Friedman test [*χ*^2^ (3) = 15.10, *p* < 0.005] followed up with *post hoc* Bonferroni corrected Wilcoxon tests (alternative hypotheses accepted if *p* < 0.0083) showed that the number of regressive eye movements was significantly higher for poetic reappraisal than prosaic non-reappraisal (*T* = 0, *p* < 0.005, *r* = −0.91, one-tailed). No other significant differences between text types were found in number of regressive eye movements (see Figure 2).

**Figure 3:**
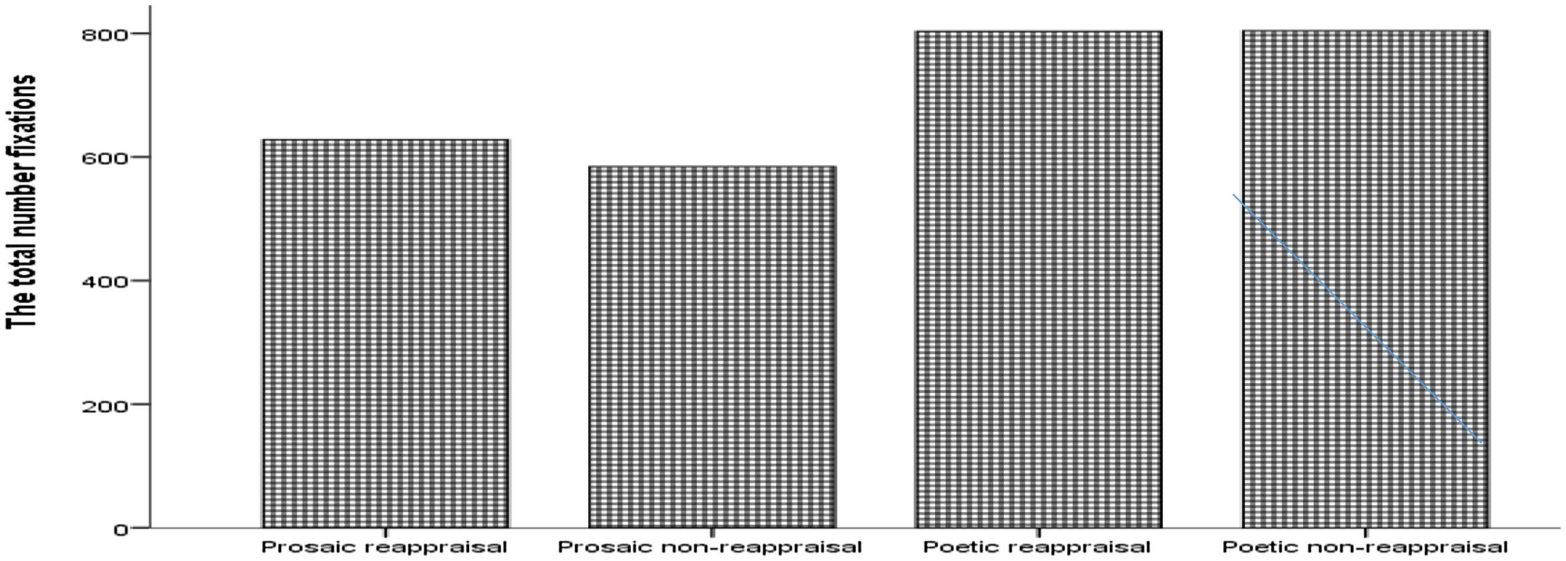
Bar graph showing the total number of fixations by text type.

When considering the number of fixations, although the overall Friedman test *χ*^2^ (3) = 14.67 *p* < 0.005 was significant, none of the *post hoc* Wilcoxon tests reached significance after Bonferroni correction (See Figure 3).

**Figure 4:**
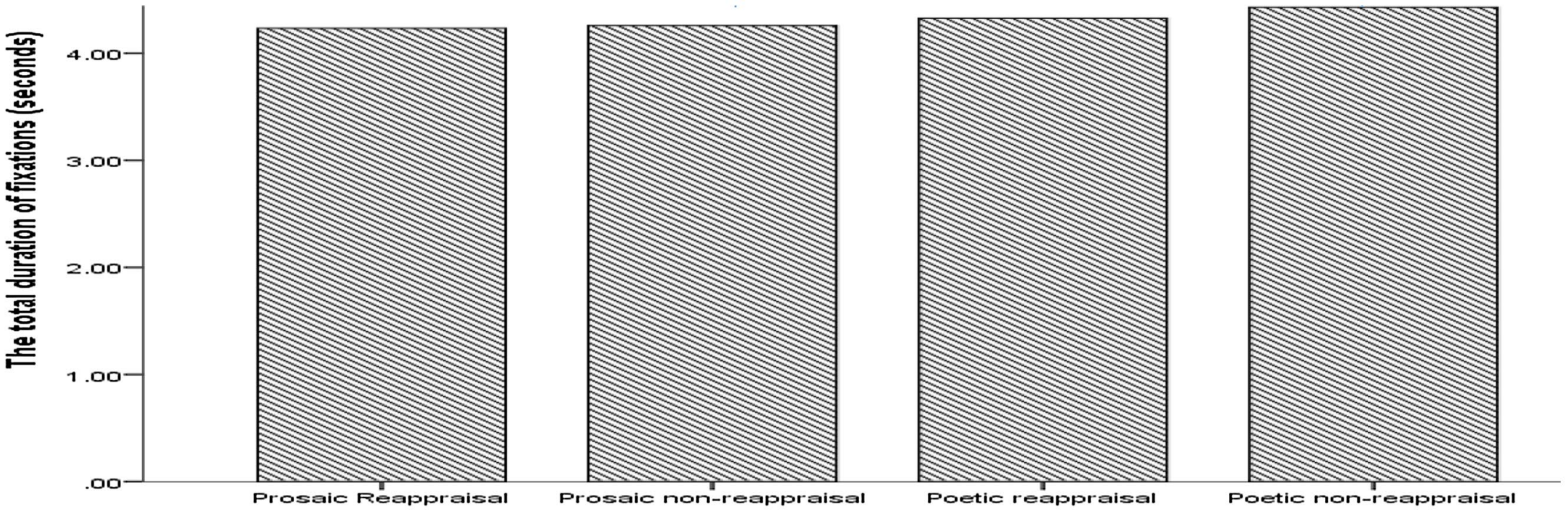
Bar graph showing the total duration of fixations by text type. Poetic reappraisal > prosaic non reappraisal; *p* = 0.004.

A significant Friedman test χ^2^(3) = 14.48, *p* < 0.005 with *post hoc* Bonferroni corrected Wilcoxon tests showed that the duration of fixations was significantly higher for poetic reappraisal than for prosaic non-reappraisal (*T* = 15, *p* < 0.005, *r* = −0.70, one-tailed). No other significant differences were found in terms of duration of fixations (See Figure 4).

### Association with literary familiarity

The median score of the sample on the literary familiarity measure was 4 with a range of 10. A series of two tailed Spearman’s correlations exploring the relationship between self-reported literary familiarity and eye tracking metrics were non-significant (number of regressions across all text types *r* = 0.13, *p* = 0.62; total number of fixations across all text types *r* = 0.41, *p* = 0.12; duration of fixations across all text types *r* = −0.16, *p* = 0.56).

## Discussion

The current study investigated the reading of 4-line texts which were either poetic or prosaic in nature and which either did or did not embed an explicit need to reappraise the text’s meaning at the final line. Using simple eye tracking metrics, the study aimed to explore ideas originally expounded by Coleridge (1817) that the reading of poetic texts is not linear but rather involves a complex back and forth revisiting of words and areas within the text that demand different levels of, or further, processing by the reader. To do this the analysis focussed on the number of regressive eye movements recorded and the number and length of fixations within the short texts. It was anticipated that the reading of poetic texts would be characterised by higher values of all metrics compared to prosaic texts. Self-reported familiarity with literary material was also considered in relation to these eye tracking metrics where differences in the reading patterns of participants were anticipated to be associated with reported familiarity with poetic texts.

The findings showed that poetic texts did prompt significantly more regressive eye movements as well as more and longer fixations compared to prosaic texts, supporting Coleridge’s hypotheses.

It was further shown that regressive eye movements did likely reflect the need to re-or further appraise the meaning of the short texts because their frequency was significantly greater for those texts that embedded the need for major reappraisal in the last line compared to those that did not. The nature of the last line did not affect the fixation variables, however. The Friedman analyses comparing all text types illustrated that the major differences between the text types lay in the number of regressive eye movements and the duration of fixations when poetic reappraisal pieces were compared with prosaic non-reappraisal pieces.

The overall pattern is in keeping with the suggestion that poetic pieces, especially if they require major reappraisal at the final line, are associated with deeper, slower reading compared to prosaic pieces that do not require major reappraisal at the final line. The fact that poetry compared to prose prompts more regressive eye movements as well as longer fixations is consistent with poetry requiring deeper consideration and appraisal of meaning, suggesting more cognitive effort to achieve congruence with the reader’s situational model (McNamara et al., 1996). Indeed, maybe the unpredictable nature of poetic texts, evidenced here in the eye tracking metrics, and perhaps experienced by the reader as a series of valid surprises, has potential to alter or update the real-world situational model.

Contrary to expectations, self-reported literary familiarity was not associated with these simple eye-tracking metrics however. This may be explained by the fact that all participants in this small sample were broadly unfamiliar with literary reading and so all could be regarded as novice readers of poetry. Experts in literary reading may show different eye tracking patterns (Reingold and Sheridan, 2011) but determining the reading patterns of such experts would require further eye tracking research.

It is argued that the findings of this study reflect the greater agility of mind needed to consider poetry compared to prose. By and large, it is safe to suppose that a more agile or flexible mind is more capable of accurately processing the complexity of real-world human experiences. If the enhanced experience of mental agility nurtured by the reading of literature can generalise in ways capable of boosting the processing of real-world episodes, then the findings reported here may illustrate positive real-world impacts of reading serious literary material. The idea that the effortful processing of rich literary texts has potential to enhance everyday human functioning is akin to mental muscle type arguments as well as the broadly accepted relationship between analogous reasoning and intelligence. In this, it is resonant of Coleridge’s suggestion that the act of literary reading is a good model for the deepest level of human thinking where habitual responses are queried, automaticity disabled and where perhaps the usually efficient reduction of rich information is disfavoured.

This study was a small scale proof of concept of Coleridge’s ideas about the literary reading brain and, as such, it has limitations not least the small and homogeneous sample of participants included who were mostly female. Attempts to increase sample size were affected by difficulties associated with the collection of data of high enough quality which meant that responses from 11 additional participants enrolled into the study were not useable. The reasons were several but included variation in the eyes of the participants that made them difficult to track consistently, some failures of procedure during testing arising from both equipment and human error and because the task was cognitively demanding and so impacted by individual differences in reading speed. These issues would need to be accounted for better in future eye tracking research examining these complex text types. A larger sample may have enabled the use of more powerful and parsimonious statistical analysis that would likely provide more convincing and definitive results.

Participant fatigue is a significant issue to consider when repeated measures are used during an intense, demanding task. For this reason, stimuli were presented in randomised order to spread any impact of fatigue evenly through the conditions. Mind wandering is a further potentially troublesome issue with designs such as this. However, as the texts were cognitively demanding, the likelihood of significant mind wandering was reduced. Furthermore, as with fatigue effects, randomisation of order should have spread any potential issues with mind wandering evenly across the conditions.

Unlike with Müller et al.’s (2017) Haikus, this study did not include a measure that assessed the extent to which participants felt they understood the pieces of text. Understanding of poetry is a matter of individual difference where people are bound to respond to and ‘get’ texts in distinct personal ways according to life experiences and preferences. Of course, this means that the objective measurement of meaning-making is challenging for these texts. Future research should attempt to assess and control for any differences between the text types in terms of the extent to which they were felt to be comprehended as poor comprehension of particular stimuli would likely result in a higher frequency of regressive eye movements, known to be related to comprehension (Schotter et al., 2014; Jarodzka and Brand-Gruwel, 2017). Furthermore, this study only explored one individual difference measure, namely participants’ subjective familiarity with poetry. While this variable was not significantly associated with eye tracking indices, it is possible that other cognitive skills such as fluid intelligence, problem-solving, analogous reasoning skills as well as tolerance of uncertainty could have influenced findings (O’Sullivan et al., 2015; Hitsuwari and Nomura, 2022a,b). In any future larger scale research these variables would be worthy of consideration. Finally, the stimuli were short sections of text only. While these were well controlled and validated by O’Sullivan et al. (2015), they are limited in the extent to which their processing can really model the reading of poetry versus prose or other literary material. Further eye tracking research is needed to reflect the different reading patterns associated with poetry, literary fiction and prosaic forms.

Any future research should consider if it is more useful to use mobile eye-tracking units as used here or more restrictive mounted headrest eye tracker kits. The latter may compromise ecological validity and participant comfort while providing data less beset by procedural challenges. With further development of mobile eye tracking equipment to make it more portable and less prone to movement artefact etc. future research, could improve our understanding of the positive power of literature by taking headsets into real world reading scenarios to sample solitary compared to more social forms of reading, for example.

The current study has shown how simple eye tracking variables can inform about reading styles and the processing of different types of text. It is possible that eye tracking and other psychophysiological metrics could be used to select reading material to objectively determine the extent to which reflective, literary processing is drawn on by different texts. This would have implications for the pedagogy of English literature. Perhaps more importantly, this type of study could advance the scientific study of the benefits of the Arts to the development of the thinking mind, replacing its value at the heart of society.

## Data availability statement

The original contributions presented in the study are included in the article/supplementary material, further inquiries can be directed to the corresponding author.

## Ethics statement

The studies involving human participants were reviewed and approved by the University Research Ethics Committee, University of Liverpool. The patients/participants provided their written informed consent to participate in this study.

## Author contributions

All authors listed have made a substantial, direct, and intellectual contribution to the work and approved it for publication.

## Conflict of interest

The authors declare that the research was conducted in the absence of any commercial or financial relationships that could be construed as a potential conflict of interest.

## Publisher’s note

All claims expressed in this article are solely those of the authors and do not necessarily represent those of their affiliated organizations, or those of the publisher, the editors and the reviewers. Any product that may be evaluated in this article, or claim that may be made by its manufacturer, is not guaranteed or endorsed by the publisher.

## Footnotes

^1^ https://pupil-labs.com

^2^ https://puredata.info
